# Practical Approaches to Apply Ultra-Thick Graphite Anode to High-Energy Lithium-Ion Battery: Carbonization and 3-Dimensionalization

**DOI:** 10.3390/nano12152625

**Published:** 2022-07-29

**Authors:** Junsu Park, Seokho Suh, Sigitas Tamulevičius, Daesoo Kim, Dongin Choi, Sungho Jeong, Hyeong-Jin Kim

**Affiliations:** 1 Ground Technology Research Institute, Agency for Defense Development, Yuseong-gu, P.O. Box 35, Daejeon 34186, Korea; jspark1@add.re.kr; 2 Graduate School of Energy Convergence, Institute of Integrated Technology, Gwangju Institute of Science and Technology, 123 Cheomdangwagi-ro, Buk-gu, Gwangju 61005, Korea; seok2659@gm.gist.ac.kr; 3 Institute of Materials Science of Kaunas, University of Technology, Barsausko St. 59, LT-51423 Kaunas, Lithuania; sigitas.tamulevicius@ktu.lt; 4 LG Energy Solution, Ltd., 188 Munji-ro, Yuseong-gu, Daejeon 34122, Korea; daesookim@lgensol.com (D.K.); dichoi@lgensol.com (D.C.); 5 School of Mechanical Engineering, Gwangju Institute of Science and Technology, 123 Cheomdangwagi-ro, Buk-gu, Gwangju 61005, Korea

**Keywords:** lithium-ion batteries, thick electrodes, graphite anodes, binder carbonization, laser structuring

## Abstract

Lithium-ion batteries with ultra-thick electrodes have high energy density and low manufacturing costs because of the reduction of the inactive materials in the same battery volume. However, the partial usage of the full capacity and the low rate capability are caused by poor ionic and electronic conduction. In this work, the effects of two approaches, such as electrode binder carbonization by heat treatment and 3-dimensionalization by the laser structuring of ultra-thick graphite anodes to lithium-ion batteries for high energy density, are investigated. During the heat treatment, the polyvinylidene fluoride (PVDF) binder is carbonized to form fluorinated graphitic carbons, thereby increasing the number of lithium-ion storage sites and the improvement of the electrode capacity by 14% (420 mAh g^−1^ and 20 mAh cm^−2^). Further, the carbonization improves the rate capability by 31% at 0.1 C by simultaneously reducing the ionic and electronic resistances. Furthermore, after the laser structuring of the carbonized electrode, the areal discharge capacity increases to 50% at the increasing current rates, resulting from drastically improved ionic conduction. In addition to the electrochemical characteristics, these two approaches contribute considerably to the fast wetting of the electrolyte into the ultra-thick electrode. The carbonization and laser structuring of the ultra-thick graphite anodes are practical approaches for high-energy batteries to overcome the thickness limitation.

## 1. Introduction

Lightweight lithium-ion batteries (LIBs) with a high operating voltage and long life cycle are in demand for the success of electric vehicles and a sustainable energy economy [1,2]. Graphite has been widely used as an anode material because of its long cycle life and stability [3]. A current battery is manufactured by repeatedly stacking cells, which consist of cathode materials on the current collector (aluminum), a separator, and anode materials on the current collector (copper). Considering this internal configuration of the battery, the practical approach to enhancing the energy density of LIBs is to increase the amount of active materials by thickening the electrodes while reducing the inactive components, such as the separator and current collector, in the same volume [4,5,6]. Since the number of electrode stacks occupying the same volume can be reduced by using thick electrodes, the manufacturing cost can be reduced by decreasing the amount of inactive materials. Further, the manufacturing efficiency can be improved by reducing the processing time for cutting and stacking individual electrodes [7,8,9].

However, the utilization of the thick electrode is not a simple approach to achieving high energy density because poor ionic and electronic conductions in the thick electrode cause a partial reduction in the theoretical capacity of an LIB and the deterioration of LIB performance, such as rate capability [10,11]. For instance, Singh et al. [12] have demonstrated that a full cell produced using thick graphite (320 μm) and lithium nickel manganese cobalt oxide (NMC) electrodes shows only two-thirds of the total capacity at 0.5 C. Additionally, Lu et al. [13] have investigated the effects of lithium nickel cobalt aluminum oxide (NCA) electrodes on electrode thicknesses ranging from 20 to 150 μm. The authors revealed that the thickest electrode exhibits the poorest rate capability and only 10% usable capacity of the theoretical capacity owing to its high internal resistance. Similarly, Gallagher et al. [14] have reported that the degradation of the thick electrode performance, such as rate capability or usable capacity, results in limited capacity improvement because of the increase in the electrode thickness. The high tortuosity of the thick electrodes results in long ionic and electronic pathways, thereby increasing internal resistances, such as ionic, electronic, and charge transfer resistances [15,16,17,18]. To remarkably enhance the LIB performance, both the ionic and electronic conduction of the electrodes should be simultaneously improved [19,20,21,22,23,24].

Laser structuring has recently been applied to various LIB electrodes for better ionic diffusion within thick electrodes. Silicon/graphite composites [25], graphite [26], and NMC [27] electrodes 3-dimensionalized by the laser structuring show a significant improvement in ionic diffusion and electrolyte wetting. Thus, they considerably increase the capacity and enhance the rate capability and life cycle of the thick electrodes (100–200 μm). Additionally, to improve the electronic connection in silicon and graphite anodes, various binders, such as polyvinylidene fluoride (PVDF) [28], polyamic acid (PAA) [29], and lignin [30], have been carbonized by heat treatment at 500–800 °C. After carbonization, the homogeneously distributed binders within an electrode function as an evenly built electronic network. Since the commonly used binders have poor electrical conductivity, the carbonization of the electrode binder significantly improves the conductivity of the electrodes. In particular, the carbonization of the PVDF binder has several advantages. First, the carbonized PVDF binder has high electrical conductivity originating from the semi-ionic bonding features between carbon and fluorine. Second, it increases the number of storage sites for lithium ions because fluorinated graphite can reversibly react with lithium ions. Finally, mesopores are formed, resulting in improved electrolyte impregnation and lithium-ion diffusion in the electrodes [31]. As reported above, these approaches are applicable for improving ionic or electronic conduction in conventional or relatively thick electrodes (≤200 μm).

Unfortunately, at the level of ultra-thick electrodes (≥500 μm), their utilization is challenging owing to geometric and electrochemical characteristics, such as ionic and electronic resistance. To utilize the ultra-thick electrode for the high-energy LIBs by minimizing the inactive components at the same volume of LIBs, the simultaneous enhancement of ionic and electronic conduction is indispensable. However, the electrochemical studies on ionic diffusion and electronic conductivity in the ultra-thick electrodes are still unclear, and reports on performance are still limited, particularly for carbonization and/or 3-dimensionalization.

In this work, we report the effects of carbonization and/or laser structuring on ionic and electronic conduction and the electrochemical performance of the ultra-thick graphite anodes. Additionally, we suggest that carbonization and laser structuring are practical approaches for utilizing ultra-thick electrodes in high-energy LIBs. As evidentiary data, we analyze the electrochemical characteristics, such as rate capability and electrochemical impedance spectra, depending on carbonization and laser structuring, and compare them with a conventional thick graphite anode consisting of graphite, conductive carbon, and a binder. The geometry, surface chemistry, and bulk resistances of the individual electrodes are investigated to characterize the carbonized and/or 3-dimensionalized ultra-thick electrodes and their electrochemical performances in various aspects.

## 2. Experimental Section

### 2.1. Materials and Electrode Preparation

Graphite and Super C were obtained from MTI Corporation (Richmond, CA, USA). Polyvinylidene fluoride (PVDF, Solef 5130) was purchased from Solvay (Tuscany, Italy). Lithium foil was obtained from NEBA (Daejeon, Korea). All reagents were used without further purification.

As a reference electrode, a typical graphite anode was prepared by mixing 92 wt.% graphite, 3 wt.% conductive carbon, and 5 wt.% PVDF binder dissolved in N-Methyl-2-pyrrolidone (NMP) using a planetary ball mill (PM100, Retsch Technology, Haan, Germany) with a rotation speed of 150 rpm for 30 min under an argon atmosphere. The mixed slurry was coated on Cu foil (thickness 20 μm) with a doctor-blade at a rate of 9 cm/s; the gap between the edge of the doctor-blade and the foil was 1.1 mm. The coated electrode was dried at 100 °C in an oven for 30 min and then kept in a vacuum for 12 h. Next, electrodes were uniformly calendared using a roll press machine. Before and after calendaring, the thicknesses were 520 μm and 410 μm; the porosity was about 50% and 39%, respectively. The porosity of electrodes is defined as follows:

(1) $$\mathrm{Porosity}\;\left(\%\right)=\frac{V-W\;\left\{\;\frac{C_{1}}{\rho_{1}}+\frac{C_{2}}{\rho_{2}}+\frac{C_{3}}{\rho_{3}}\;\right\}}{V}\times 100$$

where *V* is the volume of the composite electrode without a current collector, *C* is the proportion of each material in the electrode composite, *W* is the weight per area, and *ρ* is the density of each material. For carbonization and laser structuring, electrodes, which were composed of 92 wt.% graphite and 8 wt.% PVDF binder except for the conductive carbon, were fabricated with the same procedure. The loading masses of graphite active material in the entire electrodes were about 49 ± 1 mg cm^−2^. The NCM electrodes were composed of 93 wt.% NCM active materials, 3 wt.% conductive carbon, and 4 wt.% PVDF binder. The n/p ratios in NCM/Gr full cells were set to around 1.05.

### 2.2. Carbonization and Laser Structuring

Note that the naming of electrodes was designated as follows: typical electrode (consisted of graphite, PVDF binder, and conductive carbon), original electrode (consisted of graphite, PVDF binder, and no conductive carbon), carbonized electrode as PVDF-C, and carbonized laser-structured electrode as PVDF-C-L. To prepare PVDF-C, the electrode was heated from ambient temperature to 600 °C for 1 h at a heating rate of 2 °C min^−1^ under continuous argon flow using a tube furnace.

Ultra-short pulsed laser (PH1-sp-1.5, light conversion; Yb:KGW laser, pulse duration = 190 fs, repetition rate = 30 kHz, maximum power = 6 W at a wavelength of 515 nm) was used for the laser structuring of graphite anode. For high-speed processing and simplicity of application, straight grooves were produced at the electrode surface during laser ablation using a galvanometer scanner (SCANLAB) with a focusing lens (focal length = 167 mm). From pretests, laser structuring parameters such as pulse energy, groove depth and pitch were determined by considering thermal damage and material loss by laser ablation. When pulse energy exceeded 30 μJ, melting and agglomeration of partial materials occurred. Therefore, the reasonable laser pulse energy of 30 μJ was determined to achieve little thermal effects and high processing speed. At this laser energy value, groove depth and pitch were selected to be 200 μm (half of the electrode thickness) and 200 μm, respectively. The groove width was approximately 40 μm at the electrode surface, and the number of scans to reach the desired depth was seven. The scan speed was applied at 18 mm s^−1^. The electrodes were held on a multi-axis stage by a vacuum chuck, and the generated particles during laser structuring were removed by air blowing.

### 2.3. Cell Assembly and Electrochemical Analysis

For half-cell tests of coin type, all electrodes were punched with a diameter of 10 mm without delamination between the electrode material and a current collector. They were assembled into a CR2032 coin cell in an argon-filled glove box with oxygen and water contents less than 0.5 ppm. A polypropylene membrane (Celgard 2400, thickness = 20 μm, diameter = 19 mm) was used as the separator, and lithium foil (thickness = 0.4 mm, diameter = 17 mm) was used as the counter electrode and reference electrode. The electrolyte contained 1 M LiPF_6_ in ethylene carbonate (EC)/diethyl carbonate (DEC) (1:1, *v*/*v*). After filling the electrolyte, a slight vacuum was formed by a vacuum pump for a few seconds to impregnate the electrolyte into the electrode pore. After assembly, the cells were stabilized at room temperature for 12 h for further wetting.

For evenly formed SEI layers, slow formation cycles of the assembled cells were conducted at 0.02 current rate (C-rate) in a voltage range from 0.01 to 1.0 V. The galvanostatic charge/discharge performance was analyzed using a battery cycler (WBCS 3000, Wonatech, Korea) while increasing current rate from 0.02 C to 0.3 C. In all electrochemical tests, five samples for each type were used for reproducibility. An EIS analysis of the electrodes was carried out to measure the ionic resistance (passing through pores in electrodes) at the voltage amplitude of 10 mV in the frequency range from 500 kHz to 5 mHz using a potentiostat (VSP, Biologic, France). Herein, as-prepared electrodes were used to simplify the impedance analysis by removing a charge transfer resistance (R_ct_) that overlapped with the impedance spectrum of an ionic resistance (R_ion_). In particular, we assembled symmetric cells, which consisted of two identical electrodes, to analyze the intrinsic impedance characteristics of individual electrodes. The Z-fit program (Biologic) was used to fit the Nyquist impedance plots and to calculate the internal resistance values. Modified restricted diffusion (MRD) was considered for the fitting, and a mathematical expression for the impedance analysis was referred to [32,33]. The tortuosity values were calculated based on Equation (2) using results of ionic resistance values [34,35].

(2) $$\tau =\frac{R_{ion}\cdot A\cdot k\cdot \varepsilon }{2t}$$

where *A* is the electrode surface area, *κ* is the conductivity of the electrolyte, *ε* is the electrode porosity calculated by Equation (1), and *t* is the electrode thickness.

To compare the electrochemically active surface area (ECSA) of thick Gr electrodes, the cyclic voltammetry (CV) test was employed using a non-lithium intercalating electrolyte, 0.5 M tetrabutylammonium perchlorate (TBAClO_4_, Sigma-Aldrich, St. Louis, MO, USA, >99%) in EC/DMC (1:1, *v*/*v*) to prevent a faradaic current. The CV measurement was performed at open circuit potential (OCP) to identify the electrode capacitive currents. The potential range was 50 mV at scan rates from 1 mV s^−1^ to 10 mV s^−1^. The specific capacitance was calculated as follows [36,37].

(3) $$C_{sp}=\frac{I_{c}-I_{d}}{s\cdot m}$$

where *C_sp_*_,_ is the specific capacitance (F g^−1^), *I_c_* and *I_d_* are cathodic and anodic current density (mA g^−1^), *s* is the scan rate (mV s^−1^), and *m* is the active material mass of an electrode (g).

### 2.4. Electrode and Material Characterizations

To analyze the morphology of electrodes before/after laser structuring and/or binder carbonization, a scanning electron microscope (FE-SEM, HITACHI) was used. An electronic scale (ML-204, Mettler Toledo, resolution = 100 μg) was used to measure the mass of electrodes to check the mass changes resulting from carbonization and laser structuring. The change of interfacial area between electrolyte and electrode surface layer newly revealed by laser structuring was analyzed by using a 3D confocal microscope (VK-X200K, KEYENCE, Itaska, IL, USA).

Chemical elements at the surface of typical and heat-treated graphite electrodes were investigated by X-ray photoelectron spectroscopy (XPS, K-Alpha, Thermo Fisher Scientific) using a micro-focused monochromated Al Kα X-ray source (measuring diameter of 200 μm) under vacuum of 5 × 10^−9^ mbar. All spectra were acquired and processed by using Thermo Avantage software. An electrode resistance meter (HIOKI, XF057) was used to analyze the improvement of electrical conductivity after the carbonization of a conventional graphite anode.

Fourier-transform infrared (FTIR) spectroscopy was conducted to confirm the structure of the PVDF binder before and after heat treatment. FTIR spectra were collected using a Vetex70v FTIR spectrometer at room temperature in the range of 650–4000 cm^−1^, with attenuated total reflectance (ATR) mode. Raman data was acquired by a Raman spectroscopy (LabRAM HR Evolution, Horiba Jobin Yvon, Longjumeau, France) with a 532 nm laser (laser power was about 0.7 mW) at ambient room temperature conditions.

To investigate the wetting characteristics of carbonized laser-structured and conventional graphite anodes, an optical contact angle meter (DSA25, KRUSS GmbH, Hamburg, Germany) was used to measure contact angles on an electrolyte droplet on the electrodes. For the measurement, the electrodes were fixed on a slide glass, and then a 3.5 μL electrolyte droplet was dropped on the electrode surface. Digital images were collected using high speed camera (200 fps), and the tangent fitting type was applied for the measurement of the contact angles. The same measurement was repeated five times on each electrode, and the average was used for the contact angle.

## 3. Results and Discussion

### 3.1. Physicochemical Characteristics of the Electrode

**Figure 1:**
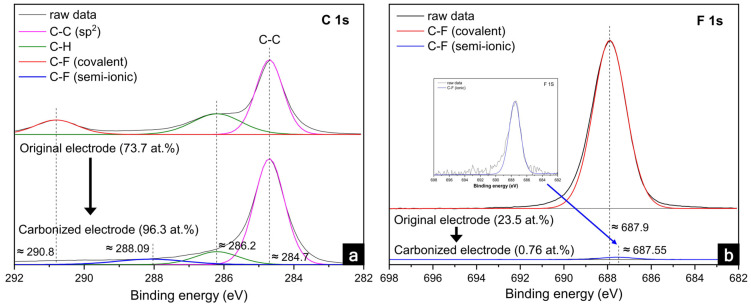
XPS data of (**a**) C and (**b**) F before and after the carbonization of the graphite anode.

To analyze the carbonization of the PVDF binder, the chemical elements at the electrode surface were investigated using X-ray photoelectron spectroscopy (XPS) measurements. The general profiles of the XPS spectra of the graphite electrodes are shown in Figure 1, and Table 1 lists the individual atomic percentages before and after heat treatment. For the original electrode shown in Figure 1a, the XPS spectra of C 1s were divided into three peaks at 284.7, 286.2, and 290.8 eV. Among them, 284.7 eV corresponded to the sp^2^-hybridized carbon (C–C) of the active materials of graphite [38]; 286.2 eV was attributed to carbon atoms single-bonded to hydrogen, and 290.8 eV was associated with the covalent C–F bond of the PVDF binder [39]. After the heat treatment, the C 1s peak at 284.7 in the carbonized electrode was the same as that of the original electrode, indicating that the graphite was not chemically affected during the heat treatment. Conversely, the C–F (covalent) peak at 290.8 eV disappeared, as shown in Table 1. Instead, a peak at 288.09 eV indicating a C–F (semi-ionic) bond emerged [40]. For the F 1s spectra shown in Figure 1b, the peak at 687.9 eV corresponding to the C–F (covalent) bond vanished [41]. Meanwhile, another peak at 687.55 eV associated with the C–F (semi-ionic) bond was revealed as the change in the C–F peaks (covalent and semi-ionic) in C 1s [42]. After the heat treatment, the sp^2^ carbon composition increased; however, the C–H and C–F bonds decreased owing to the cyclization of the PVDF and the release of HF from the carbon chain in the PVDF [28].

**Figure 2:**
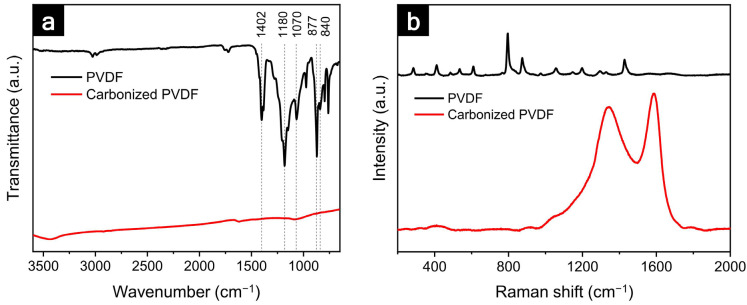
(**a**) FTIR spectra and (**b**) Raman spectra of pristine and heat-treated PVDF powders.

For further chemical analysis, Fourier-transform infrared (FTIR) and Raman spectroscopies were performed on the as-prepared PVDF and carbonized PVDF powders (Figure 2). In the FTIR spectrum of the PVDF, the peaks at 1402 cm^−1^ and 1070 cm^−1^ corresponded to CH_2_ wagging; the peaks at 1180 cm^−1^ and 840 cm^−1^ were associated with the C–F stretching; the C–C–C asymmetric stretching of the PVDF was observed at 877 cm^−1^ [43]. However, no noticeable peaks were observed in the carbonized PVDF, indicating the decomposition of the C–F and C–H bonds during the thermal treatment. The Raman spectrum of the carbonized PVDF showed two broad peaks at 1342 cm^−1^ and 1586 cm^−1^ that were assigned to the D and G bands, respectively [44]. The G band was evidence of the in-plane bond stretching of the sp^2^ carbon atoms, and the intensity ratio (*I_D_*/*I_G_*) was 0.913, demonstrating the graphitization of the heat-treated PVDF powders.

**Figure 3:**
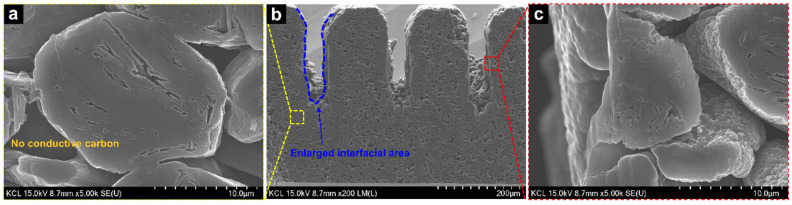
Cross-section SEM images of an original electrode at magnifications of (**a**) 200×, (**b**) 1000×, and (**c**) 5000×.

**Figure 4:**
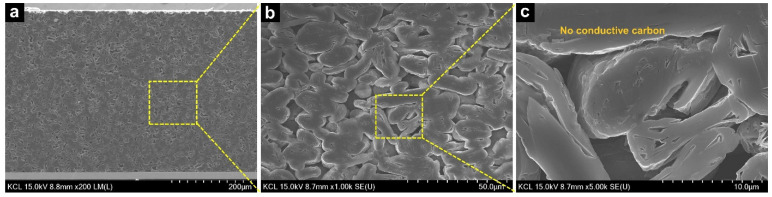
Cross-section SEM images of the PVDF-C-L electrode at magnifications of (**a**) 5000×, (**b**) 200×, and (**c**) 5000×.

In addition to the chemical analysis, the physical and morphological characteristics were analyzed using scanning electron microscopy (SEM) images, and the mass of the electrodes was changed. As observed in the typical thick graphite electrode shown in Figure S1, a large amount of conductive carbon was distributed around the graphite. Since the nano-sized conductive materials were covered with the graphite and were densely located among the active materials, few pores were used for lithium ions to smoothly diffuse within the electrode. In contrast, Figure 3 reveals relatively large pores among the active materials in a PVDF-C because the electrode consisted of only graphite particles and partially carbonized PVDF binders without conductive carbons. The large pores in the PVDF-C were believed to improve the impregnation and ionic conductivity of the electrolyte. Figure S2 shows the surfaces of the original and PVDF-C-L electrodes. No significant defects, such as electrode cracks, bulges, or voids, were observed in the electrodes. Figure 4 shows the cross-sections of the PVDF-C-L electrode. As shown in Figure 4c, it was observed that the pores among the active materials were not influenced by the laser structuring. Further, the cross-sectional images showed that the individual graphite particles were precisely cut without melting by laser processing, and the groove depth was approximately half of the electrode thickness. After the laser structuring, the interfacial area between the electrolyte and electrode surface, including the laser-structuring regions, increased by approximately 350% (original = 252.4 mm^2^ vs. PVDF-C-L = 1136.5 mm^2^).

After the heat treatment, the weight of the original electrode decreased by approximately 5 wt.%. Since graphite was stable at 600 °C, it was assumed that the weight loss results from the disappearance of H and F atoms in the PVDF binders rather than from of the active materials. The electrode mass due to the laser structuring of the PVDF-C decreased by approximately 14 wt.% (PVDF-C = 49 mg cm^−2^ vs. PVDF-C-L = 42 mg cm^−2^).

### 3.2. Electrochemical Characteristics of the Electrode

**Figure 5:**
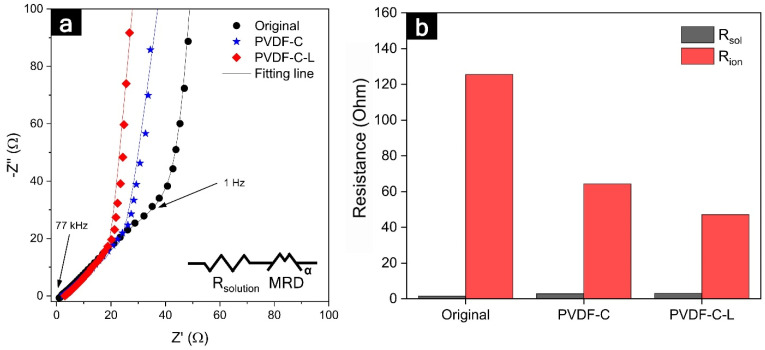
(**a**) EIS data of symmetric pristine electrodes, depending on the carbonization and laser structuring; (**b**) comparison of each resistance component in the graphite electrodes.

The internal resistances of the original electrodes were affected by the carbonization and laser structuring, as shown in Figure 5. Typically, a semicircle in the high-frequency range is related to the electronic resistances, such as the contact resistance between the electrode materials and the current collector, as well as among the particles composing the electrode. Subsequently, a linear slope (45-degree angle from Z’-axis) in a low-frequency range was correlated with the ionic resistance penetrating the electrolyte within the pores of the electrode. However, a semicircle at the high-frequency range was not observed in the electrochemical impedance spectroscopy (EIS) data of the original and PVDF-C-L electrodes. We believe that this was due to the outstanding electronic conductivity of the conductive carbons and semi-ionic C–F bonding among the graphite particles in the original and PVDF-C-L electrodes, respectively. Similarly, the excellent electronic network of graphite and lithium titanate anodes in a previous study afforded a very low electronic resistance, thereby showing no semicircles in either electrode in the high-frequency range [45]. Although it seemed small enough to be difficult to discern the electronic resistances in the EIS data, the electronic resistance in the PVDF-C-L (=2.9 ± 0.1 mΩ cm^−2^), measured using an areal resistance meter, was reduced by 90%, compared with that of the original electrode (=28.5 ± 2.7 mΩ cm^−2^). In the XPS data shown in Figure 1, the carbon atoms with the semi-ionic C–F bonds are characterized by the sp^2^ carbon, which has high electrical conductivity, while the carbon atoms with the covalent C–F bonds are characterized by the sp^3^ carbon, which has poor electrical conductivity [46,47]. Additionally, the F incorporated in the C could act as an electron donor, which could further enhance the conductivity [48]. Therefore, the carbonization of the PVDF binder was significantly beneficial for improving the electronic network within the electrode, despite the exclusion of conductive agents. Additionally, the areal electronic resistances of the PVDF-C-L and PVDF-C were not compared because the laser structuring changed the bulk morphology of the carbonized electrodes, which were sufficiently conductive.

In the low-frequency range, ionic resistances were dominant for carbonization and laser structuring. As indicated in Figure S1, because the conductive carbons are densely positioned among the active materials in the original electrode, it is plausible that the conductive additives interrupted the lithium-ion diffusion within the electrode, thereby causing the ionic diffusion pathways to appear significantly tortuous [49]. However, it is understood that the morphology of the carbonized electrode is advantageous for ionic diffusion because the conductive carbons, which would cause winding diffusion paths, were not included, and a portion of the binder was vaporized during the heat treatment. Therefore, the ionic resistance value of the PVDF-C electrode was 49% lower than that of the original electrode (125.5 → 64.3 Ω). Further, the laser structuring resulted in a 27% reduction (64.3 → 47.1 Ω) in the resistance of the PVDF-C electrode. We believe that this was related to the enlarged and shortened ionic diffusion pathways. The interfacial area for the ionic diffusion between the electrode surface and electrolyte in the original electrode was enlarged by 350% as a ‘highway’ (the blue dot-line in Figure 4b). Further, the ionic diffusion distance from the electrode surface to the current collector was reduced by 200 µm, which was the same for the groove depth. Moreover, it was considered that the improvement in the ionic resistance was similar to the merit of relatively thin and less-tortuous electrodes than the ultra-thick electrode used in this study. Since the tortuosity was inversely proportional to the porosity, as presented in Equation (2), the considerably porous electrode structure contributed to the reduction in the tortuosity, thereby enhancing the performance of the LIB, such as high energy and high power densities, by improving the ionic diffusion [50,51]. It was accepted that the porosity increased in the laser-structured electrode because the laser-structured regions were filled with electrolytes that functioned as large pores. As shown in Table 2, the tortuosity values of the original, PVDF-C, and PVDF-C-L anodes were 6.8, 3.9, and 3.37, respectively, demonstrating the shortest ionic transport pathway in the PVDF-C-L anode. Owing to these morphological benefits of the laser structuring, the significantly decreased ionic resistance in the carbonized electrode can be decreased by one more step.

**Figure 6:**
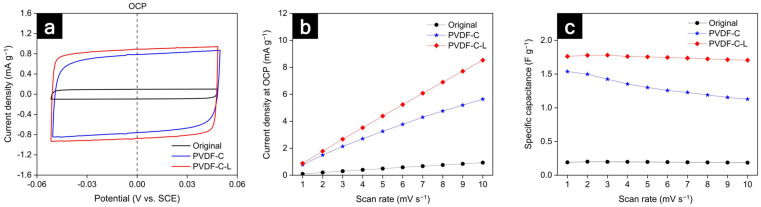
(**a**) CV curves at a scan rate of 1 mV S^−1^; (**b**) the current densities in the OCP state versus the scan rate; (**c**) the variation of specific capacitance versus scan rate.

To compare the electrochemically active surface area (ECSA) of the graphite electrodes, the non-aqueous electrochemical double-layer capacitance (DLC) was analyzed using cyclic voltammetry (CV) measurements, as shown in Figure 6. Instead of an electrolyte containing lithium salts, a non-lithium intercalating electrolyte was used to provide reasonable estimates of the ECSA by preventing faradaic current. As shown in Figure 6b, the current densities in the open circuit potential (OCP) state indicate the approximations of the area of each CV curve, which are proportional to ECSA [52]. As shown in Figure 6c, the C_sp_ values of the original, PVDF-C, and PVDF-C-L electrodes at a scan rate of 1 mV s^−1^ were 0.189, 1.53, and 1.75 F g^−1^, respectively. Since the capacitance values were proportional to the ECSA, the binder carbonization and laser structuring have been demonstrated to significantly increase the electrode surface area.

**Figure 7:**
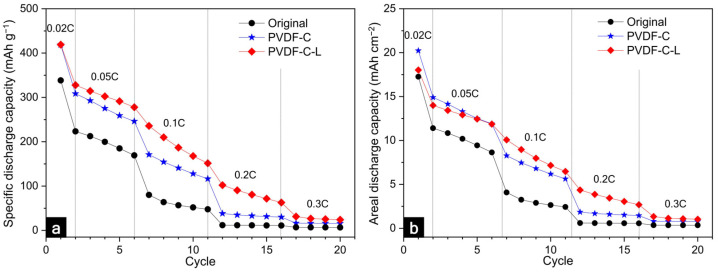
Comparison of the performances, such as (**a**) the discharge capacity normalized by the mass of the active material and (**b**) the areal discharge capacity.

To investigate the effect of the carbonization and laser structuring on the LIB performance, the specific discharge capacity normalized by the electrode mass and areal discharge capacity were measured for the current rates. As shown in Figure 7a, the specific discharge capacity of the original graphite anode showed the theoretical capacity of the conventional graphite anode (372 mAh g^−1^) at 0.02 C. However, at 0.05 C, the discharge capacity of the original electrode significantly decreased, and thereafter, the performance gradually decreased at the elevated current rates. In PVDF-C and PVDF-C-L, the fading performance was similar to that of the original electrode at high current rates. This was mainly caused by the charge transport limitation dominated by the poor ionic diffusion and low electronic conduction in the thick electrode. Additionally, as the cycle proceeded, the accumulated diffusion limitation resulted in the reaction inhomogeneity and high resistances within the thick electrode. Further, this can lead to the degradation of the local material and the fading of the capacity [53].

Although the performances of all the electrodes were degraded at the high current rates, some differences were evident in PVDF-C and PVDF-C-L compared to the original electrode. First, the specific discharge capacities of PVDF-C and PVDF-C-L were 13% higher (approximately 420 mAh g^−1^) than that of the original graphite anode at a low current rate of 0.02 C. This resulted from the increase in the C–C peak (sp^2^) because of the carbonization of the PVDF binder. The graphitic carbon resulting from the binder carbonization can partially store lithium ions [54]. Second, the specific discharge capacity of the PVDF-C electrode increased by 31% and 14% at 0.1 C and 0.2 C, respectively. As revealed in the XPS data and discussion of the internal resistances, it was understood that the morphological and chemical characteristics improved by the carbonization contributed to the enhancement of the electronic network and relatively smooth ionic diffusion. Unfortunately, no enhancement of the rate capability was observed above 0.2 C, despite the carbonization; however, after the laser structuring of the carbonized electrode, an enhancement of the performance was revealed even at the high current rates. The specific discharge capacities of PVDF-C-L were 35%, 75%, 172%, and slightly higher than those of the original electrode at 0.05 C, 0.1 C, 0.2 C, and 0.3 C, respectively. In a previous study [55], although the local carbonization at the laser-structured regions was slightly induced owing to the thermal effect during the laser structuring, it was plausible that the electronic conduction in the carbonized electrode was slightly influenced by the laser structuring because a strong electronic network was sufficiently formed in advance by the carbonization. This result implied that the remarkable improvement in the performance of the laser-structured carbonized electrode at various current rates was due to the changed electrode geometry, for instance, the less tortuous, enlarged, and shortened ionic-diffusion pathways. Since the diffusion paths were narrow, long, and distorted in the thick electrode, the LIB performances can be governed by the ionic conductivity rather than the electronic property [56]. Finally, although a mass loss of 14% occurred owing to the laser structuring, the areal discharge capacity of PVDF-C-L increased to 133% at the elevating current rates, compared with that of the original electrode, as shown in Figure 7b. Considering that conventional LIB electrodes have a capacity range from 2.5 to 3.5 mAh cm^−2^ for various applications [57], the capacities of 21 mAh cm^−2^ in PVDF-C and 18 mAh cm^−2^ in PVDF-C-L were considerably high. Even though the areal discharge capacity decreased at the high current rates, the carbonized laser-structured electrode still showed a higher capacity than the conventional electrode until 0.2 C. As shown in Figure S3, both the original and PVDF-C-L electrodes show stable full cell life cycle at 0.05 C for 50 cycles. Consistent with the half-cell result shown in Figure 7, the full-cell discharge capacity of PVDF-C-L was higher than that of the original electrode. In a comparison of the usabilities of the conventional and ultra-thick electrodes, it was observed that the use of the thick electrode could be beneficial for reducing the manufacturing cost by decreasing the number of inactive materials because the number of electrode stacks occupying the same volume can be reduced using the thick electrodes. Further, the manufacturing efficiency could be improved by reducing the processing time for cutting and stacking the individual electrodes.

Additionally, the carbonization and laser structuring can contribute to the fast wetting of the electrolyte into the electrode, as well as the electrochemical characteristics, such as the rate capability and internal resistances. Since wetting is a time-consuming process that significantly influences the productivity and manufacturing cost of LIBs, fast wetting is highly desirable in the industry. In addition to the manufacturing aspect, full wetting of the electrode is essential, because insufficient wetting of the electrode surfaces can cause significant drawbacks in terms of partial capacity usage and reduced cycle life [58]. As presented in Figure S4 and Table S1, the initial contact angle of PVDF-C-L (28.92°) was smaller than that of the original electrode (34.75°). Further, it was observed that the wetting was faster in the PVDF-C-L than in the original electrode when considering the wetting time, which decreased the contact angle from 10.7° to 5.7° when the electrolyte permeated the electrode. We believe that the physical characteristics of the electrode surface resulted in improved wetting properties. As demonstrated in Section 3.1, the interfacial area between the electrolyte and electrode surface, including the laser-structuring regions, increased by approximately 350%. Moreover, it can be considered that the calculated porosity increased from 39% to 47% because the laser-structured regions were filled with electrolytes as the pores. Additionally, it was feasible that the laser structuring helped the electrolyte to reach the current collector and spread out over the entire electrode by reducing the electrolyte penetration path to the same extent as the laser-structuring depth. Further, it seemed that the lithium ions within the carbonized electrode relatively easily penetrated the pores among the graphite active materials because of the absence of conductive agents, as determined by comparing the results presented in Figure S1c and Figure 3c. According to the Washburn equation [59], the penetration speed of the liquid electrolyte into a porous structure is proportional to the square root of the cosine of the contact angles, interfacial area, and porosity. Therefore, we believe that the morphological characteristics that were advantageously changed by the carbonization and laser structuring supported the fast wetting of the electrolyte.

**Figure 8:**
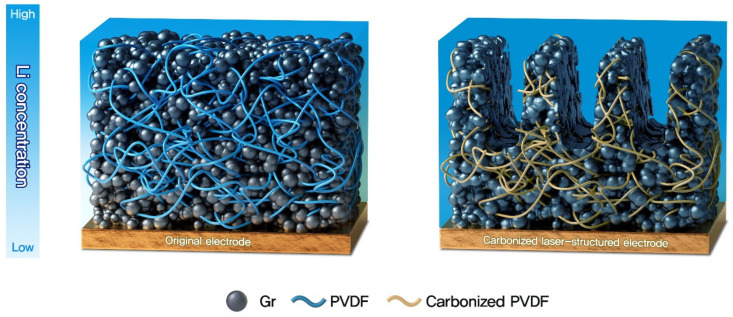
Schematic illustration of an original electrode and a PVDF-C-L electrode.

Figure 8 summarizes the explanation of the effects on the carbonization and laser structuring of the thick electrode. From the complex and dense structure of an original thick electrode, two things were inferred. First, the diffusion of the lithium ions was severely hindered because the ionic diffusion paths were tortuous. Second, since the PVDF binder served as an insulator and obstacle, it degraded the electronic network in an entire area of the electrode and the movement of the lithium ions in the electrolyte. Contrarily, the carbonization of the PVDF binders enhanced the electronic connection among the electrode materials and facilitated the smooth diffusion of the lithium ions by removing the volume occupied by the binders. Additionally, the laser structuring made the diffusion pathways of the lithium ions less tortuous and shortened them to the depth of the laser structuring from the electrode surface to the current collector. From these structural and chemical characteristics, it was observed that the thick electrodes delivered poor electrolyte wetting and ionic concentration polarization, resulting in low capacity retention at high current densities. In contrast, the PVDF-C-L electrode showed significantly enhanced electrolyte wetting and ionic flux, resulting in improved capacity retention and rate capability.

## 4. Conclusions

In this study, we investigated the effects of the carbonization of a PVDF binder induced by heat treatment and 3-dimensionalization by the laser structuring of an ultra-thick graphite anode (20 mAh cm^−2^). The carbonization of the PVDF binder contributed to the enhancement of the overall electronic network in the electrode. Since the conductive additives could be excluded from the conventional graphite anode because the electronic conduction was sufficiently enhanced by the carbonization, the ionic diffusion was improved by eliminating the conductive agents densely positioned among the active materials. Additionally, the specific discharge capacity of the carbonized electrode was increased because the mesopores formed by the carbonization offered several pores to store the lithium ions. In the laser structuring, the intended groove pitch and depth were precisely controlled by femtosecond laser irradiation with little thermal effect. Geometric changes caused by the laser structuring of the carbonized electrode resulted in enlarged, shortened, and less-tortuous diffusion pathways for the lithium ions, thereby reducing the ionic resistance in the ultra-thick electrode. This led to a considerable improvement in the areal discharge capacity at elevated current rates, despite a mass loss caused by the laser structuring. Moreover, the carbonization and laser structuring supported the fast wetting of the electrolyte into the ultra-thick electrode owing to the enlarged interfacial area and pores within the electrode. Therefore, it can be confirmed that the carbonization and laser structuring of the ultra-thick graphite anodes are practical approaches to overcoming the thickness limitation for high-energy LIBs and decreasing manufacturing costs by reducing the inactive materials and fast wetting.

## Figures and Tables

**Table 1:** Comparison of the XPS spectra for relative atomic percentages before and after the carbonization of the original graphite anode.

Peak	Binding Energy (eV)	Assignment	Original (Fraction of Species, %)	PVDF-C (Fraction of Species, %)
C 1s	284.7	C–C (sp^2^)	55.7	74.6
	286.2	C–H	27.5	20.2
	288.09	C–F (semi-ionic)	-	5.2
	290.8	C–F (covalent)	16.8	-
F 1s	687.55	C–F (semi-ionic)	-	100
	687.9	C–F (covalent)	100	-

**Table 2:** Measured and calculated parameters of the original, PVDF-C, and PVDF-C-L electrodes.

	Electrode Porosity (ε)	R_ion_	Electrode Thickness (t)	Tortuosity (τ)
Original	35%	125.5 ohm	414 µm	6.8
PVDF-C	39%	64.3 ohm	413 µm	3.9
PVDF-C-L	47%	47.1 ohm	421 µm	3.37

## Data Availability

The data that support the findings of this study are available within the article.

## Supplementary Materials

The following supporting information can be downloaded at: https://www.mdpi.com/article/10.3390/nano12152625/s1, Figure S1: Cross-section SEM images of a typical thick Gr electrode; Figure S2 SEM images of an original and a PVDF-C-L electrode; Figure S3 Comparison of full cell cycle performances such as (a) specific capacity normalized by mass of active material and (b) areal capacity of graphite anodes; Figure S4 Contact angle of an electrolyte drop at the surface of (a) an original and (b) a PVDF-C-L; Table S1 Wetting time of original and carbonized laser-treated electrodes depending on the contact angle.
