# Supplementary material for: Practical Approaches to Apply Ultra-Thick Graphite Anode to High-Energy Lithium-Ion Battery: Carbonization and 3-Dimensionalization
Source: Nanomaterials (Basel). 2022 Jul 29;12(15):2625. doi: 10.3390/nano12152625 (PMC9370293; doi:10.3390/nano12152625)
Supplement: Supplementary file 1 [file nanomaterials-12-02625-s001.zip › nanomaterials-1837923-supplementary.pdf]

nanomaterials

Article

Practical Approaches to Apply Ultra-Thick Graphite Anode to High-Energy Lithium-Ion Battery: Carbonization and 3-Dimensionalization

Junsu Park [1,†], Seokho Suh [2,†], Sigitas Tamulevičius [3], Daesoo Kim [4], Dongin Choi [4], Sungho Jeong [5,*] and Hyeong-Jin Kim [2,*]

[1] Ground Technology Research Institute, Agency for Defense Development, Yuseong-gu, P.O. Box 35, Daejeon 34186, Korea; jspark1@add.re.kr
[2] Graduate School of Energy Convergence, Institute of Integrated Technology, Gwangju Institute of Science and Technology, 123 Cheomdangwagi-ro, Buk-gu, Gwangju 61005, Korea; seok2659@gm.gist.ac.kr
[3] Institute of Materials Science of Kaunas University of Technology, Barsausko St. 59, LT-51423 Kaunas, Lithuania; sigitas.tamulevicius@ktu.lt
[4] LG Energy Solution, Ltd., 188 Munji-ro, Yuseong-gu, Daejeon 34122, Korea; daesookim@lgensol.com (D.K.); dichoi@lgensol.com (D.C.)
[5] School of Mechanical Engineering, Gwangju Institute of Science and Technology, 123 Cheomdangwagi-ro, Buk-gu, Gwangju 61005, Korea
* Correspondence: shjeong@gist.ac.kr (S.J.); hjkimc@gist.ac.kr (H.-J.K.)
† These authors contributed equally to this work.

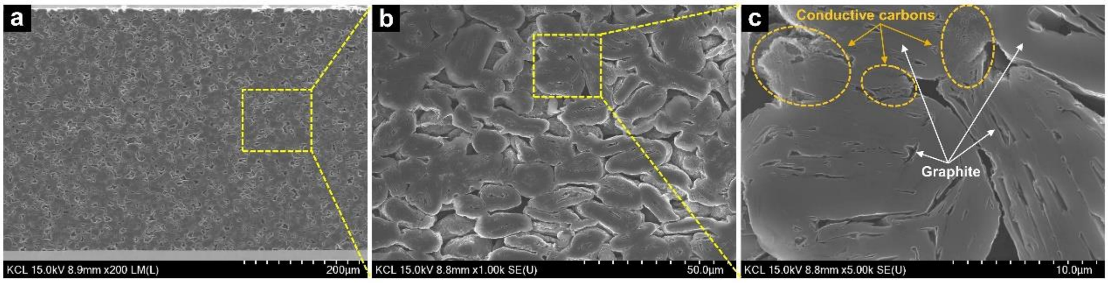

Figure S1. Cross-section SEM images of a typical thick Gr electrode.

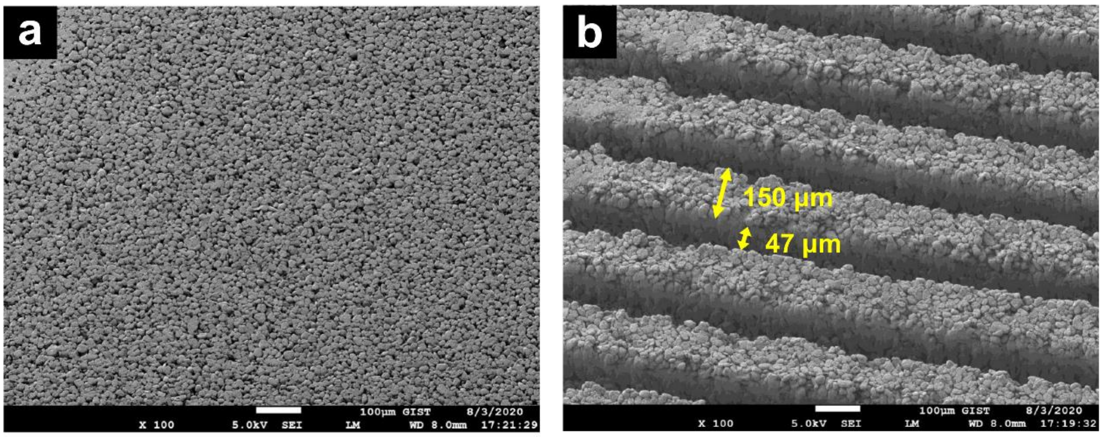

Figure S2. SEM images of an original and a PVDF-C-L electrode.

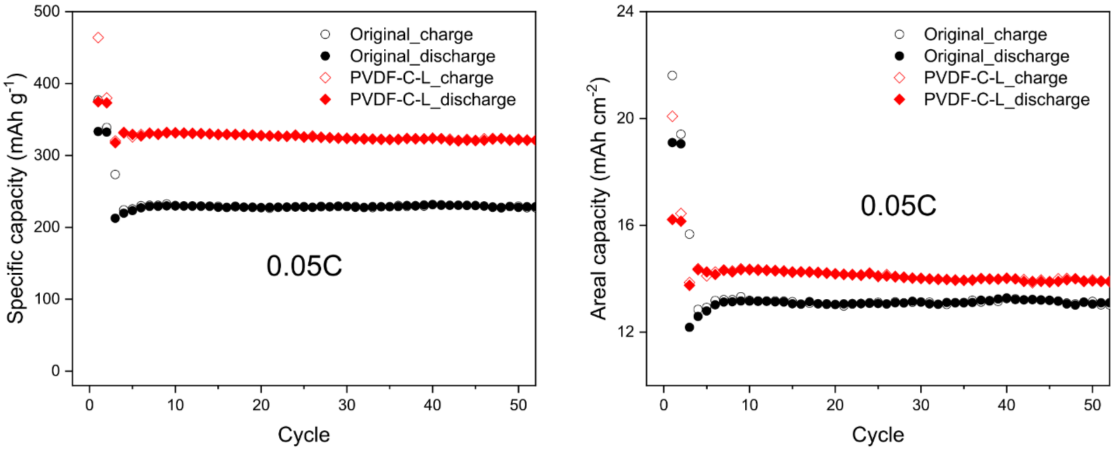

Figure S3. Comparison of full cell cycle performances such as (**a**) specific capacity normalized by mass of active material and (**b**) areal capacity of graphite anodes.

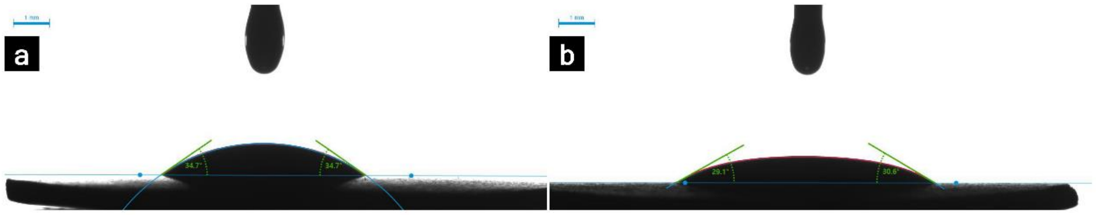

Figure S4. Contact angle of an electrolyte drop at the surface of (a) an original and (b) a PVDF-C-L.

Table S1. Wetting time of original and carbonized laser-treated electrodes depending on the contact angle.

	**Wetting time**	
	**Immediately after drop (s/20°)**	**After a while (at the same angle) (s/5°)**
Original	0.40 s (34.75° → 14.70°)	2.90 s (10.78° → 5.67°)
PVDF-C-L	0.25 s (28.92° → 8.95°)	0.20 s (10.70° → 5.71°)
